# Locus‐Specific Genetic Associations at the *DAOA* Gene in Schizophrenia and Bipolar Disorder

**DOI:** 10.1002/ggn2.202500064

**Published:** 2026-05-17

**Authors:** Madiha Khalid, Muhammad Mukhtar, Muhammad Saqlain, Abdul Sami, Shahid Mehmood Baig, Amber Nawaz, Sadaf Moeez, Ch Muhammad Fahim Ayaz, Tahzeeb Fatima, Farah Fatima, Janghoo Lim, Muhammad Nawaz, Ghazala Kaukab Raja

**Affiliations:** ^1^ Department of Health Sciences Technology National skills University Islamabad Islamabad Pakistan; ^2^ Department of Biochemistry University of Sialkot Sialkot Pakistan; ^3^ University Institute of Biochemistry and Biotechnology PMAS Arid Agriculture University Rawalpindi Rawalpindi Pakistan; ^4^ Human Molecular Genetics Laboratory Health Biotechnology Division National Institute for Biotechnology and Genetic Engineering (NIBGE) Islamabad Pakistan; ^5^ Wah General Hospital Wah Cantt. Pakistan; ^6^ Department of Biological Sciences International Islamic University Islamabad Pakistan; ^7^ Department of Chemistry and Biochemistry University of Agriculture Faisalabad Pakistan; ^8^ Department of Rheumatology and Inflammation Research Institute of Medicine Sahlgrenska Academy University of Gothenburg Gothenburg Sweden; ^9^ Department of Chemistry and Biochemistry Chemistry and Chemical Engineering Chalmers University of Technology Gothenburg Sweden; ^10^ Department of Genetics Yale University School of Medicine New Haven Connecticut USA; ^11^ Interdepartmental Neuroscience Program Yale School of Medicine New Haven Connecticut USA; ^12^ Department of Neuroscience Yale School of Medicine New Haven Connecticut USA; ^13^ Program in Cellular Neuroscience Neurodegeneration and Repair Yale University School of Medicine New Haven Connecticut USA; ^14^ Department of Biosciences and Medical Biology University of Salzburg Salzburg Austria

**Keywords:** bipolar disorder, DAOA locus, locus‐specific associations, schizophrenia, single nucleotide polymorphism

## Abstract

*DAOA* gene has been implicated in both schizophrenia (SZ) and bipolar disorder (BD), where genetic variants were associated with each disorder individually. However, the common variants shared between SZ and BD within this gene remain underexplored. The current study investigates locus‐specific genetic variants with a focus on *DAOA*. A total of 120 BD and 200 SZ patients, along with equal numbers of age‐ and sex‐matched controls from the Pakistani population, were included for genotyping, risk association analysis, haplotype, genotype‐phenotype analysis, and clinical correlations. The *DAOA* SNP rs2391191 (G/A) showed a nominal association with both SZ and BD. In contrast, rs1935062 showed no association with either disorder, but genotype‐phenotype analysis indicated nominal associations with disorder‐specific clinical features, such as insomnia and disorganized thoughts in BD, and restricted mood and feelings of threat in SZ patients. Our findings suggest locus‐specific associations for SZ and BD at the *DAOA* locus; however, due to the limited sample size, these findings should be considered exploratory and require further validation in larger cohorts. Further, patterns of allele frequencies and effect sizes diverge from several European studies and global reports, highlighting the population‐specific genetic architecture and allelic heterogeneity.

## Graphical abstract

This study explores locus‐specific genetic associations at the *DAOA* gene in schizophrenia (SZ) and bipolar disorder (BD). Using genotype, haplotype, and phenotype analyses, the authors identify a variant (rs2391191) showing associations with both disorders, while another variant (rs1935062) shows no disease association but exploratory links to clinical features. These findings highlight population‐specific genetic patterns and require further validation.

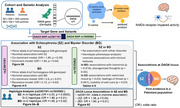

## 1 Introduction

The primate‐specific gene D‐amino acid oxidase activator (*DAOA*) is located at chromosome 13q33 and encodes the activator of D‐amino acid oxidase. *DAOA* is highly expressed in multiple brain regions. The encoded protein consists of 153 amino acids, which interacts with D‐amino acid oxidase, aiding in the oxidation of D‐serine, a critical co‐agonist of the N‐methyl‐D‐aspartate (NMDA) receptor [1]. The dysregulation of NMDA receptor signaling is reported in psychotic disorders [2], and has been strongly implicated in the pathophysiology of schizophrenia (SZ) and bipolar disorder (BD), providing a biologically plausible rationale for investigating genetic variants, i.e., single nucleotide polymorphisms (SNPs) within the *DAOA* (G72) locus [3, 4, 5, 6]. SNPs, such as rs1935062 and rs2391191, are associated with BD [7, 8, 9]. Specifically, rs2391191 (Arg30Lys) is a nonsynonymous coding variant that results in an amino acid substitution and has been among the most extensively studied polymorphisms within *DAOA* in psychiatric genetics, including SZ, BD, and related endophenotypes [3]. Functionally, both SZ and BD show reduced white matter integrity, where large‐scale genome‐wide studies (GWAS) have demonstrated a positive genetic correlation between SZ and BD [10, 11, 12, 13, 14].

Similarly, rs1935062 has been investigated in multiple psychiatric genetic studies as part of the *DAOA/G72* locus. Association studies reported a significant association between rs1935062 and bipolar disorder, supporting its potential relevance in mood disorder susceptibility [7]. Furthermore, rs1935062 has been evaluated alongside other *DAOA* polymorphisms in studies of SZ and mood disorders, and haplotype analyses spanning rs2391191 and rs1935062 have demonstrated associations with specific affective disorder subtypes, suggesting a role in phenotypic heterogeneity rather than primary disease risk [15].

While previous studies have examined variants within the *DAOA* (G72) locus in the pathogenesis of SZ and BD individually, the locus‐specific genetic association markers within this gene remain underexplored. The current study investigated whether the risk associated with rs2391191 and rs1935062 within the *DAOA* (G72) locus is shared between SZ and BD in a Pakistani cohort. Understanding these potential locus‐level genetic commonalities may provide valuable insight into the molecular basis of these psychiatric disorders, potentially informing the development of more effective treatment strategies targeting shared *DAOA*‐related pathways.

## 2 Materials and Methods

### 2.1 Ethical Approval

The current study was approved by the ethical review board of Pir Mehr Ali Shah Arid Agriculture University Rawalpindi (under the number PMAS‐AAUR/IEC‐18/2018) and the Pakistan Institute of Medical Sciences Hospital Islamabad.

### 2.2 Sample and Data Collection

The study population included 120 patients with bipolar disorder (BD) and 200 with schizophrenia (SZ), along with equal numbers of age‐ and sex‐matched controls. Written informed consent and complete medical histories were obtained from all patients and controls who participated in this study. Diagnoses of SZ and BD were made by experienced psychiatrists according to the Diagnostic and Statistical Manual of Mental Disorders, fifth edition (DSM‐5). Clinical phenotypes were systematically assessed using structured clinical interviews based on DSM‐5 symptom criteria. For all assessed variables, only the presence or absence of specific symptoms was recorded. All assessments were conducted by trained clinicians to ensure consistency and reliability.

Healthy controls were recruited from local communities and screened through a brief non‐structured interview conducted by psychiatrists. Healthy control participants with a history of mental and neurological diseases were excluded from the present study. Healthy controls were recruited from the same geographical areas as the patients. All participants were unrelated Pakistani nationals, born and residing in different areas across Pakistan.

### 2.3 Genomic DNA Extraction

After diagnosis was confirmed, 3 mL of a blood sample from each participant was collected in tubes containing ethylene diamine tetra‐acetic acid potassium salt (EDTA), using sterile syringes by an experienced phlebotomist. The blood was stored at 4°C until further processing. The genomic DNA was extracted using the standard phenol‐chloroform method [16] with minor modifications in the protocol as described in our previous study (Khalid et al., 2020) [17]. DNA quantification was performed using Thermo Scientific NanoDrop Spectrophotometer 2000. Each sample was diluted to a final concentration of 10 ng/µL prior to genotyping.

### 2.4 Genotyping

Genotyping was performed via polymerase chain reaction (PCR) using allele‐specific primers. SNP sequences were retrieved from the National Center for Biotechnology Information (NCBI) database. Allele‐specific primers were designed using BatchPrimer 3 and IDT OligoAnalyzer (Table 1). Amplification was carried out in a reaction mixture containing 10 × PCR buffer (Invitrogen, Cat. #10342020), 1.5 mm MgCl_2_, 0.2 mm dNTPs, 0.5 µm of each primer, 1 U Taq DNA polymerase (Invitrogen, Cat. #10342020), and 20 ng of genomic DNA. Thermal cycling was performed on a Bio‐Rad DNA Engine Peltier Thermal Cycler with an initial denaturation at 95°C for 5 min, followed by 30 cycles of denaturation at 95°C for 30 s, annealing at 58°C for 30 s, and extension at 72°C for 45 s, with a final extension at 72°C for 5 min. PCR products were resolved on a 1.5% agarose gel.

**TABLE 1:** Sequence of allele‐specific primers used for genotyping of *DAOA* SNPs rs2391191 and rs1935062.

Gene/SNP	Allele‐specific primers
*DAOA*/rs2391191‐Forward	ATCTACTTCATAGGTTTTCAGAG
*DAOA*/rs2391191‐Reverse	AGATTTGCTCAGAAGAATGCCCT
*DAOA*/rs1935062‐Forward	TTTGAAATCCAATCATTTTTATTTAAGAC
*DAOA*/rs1935062‐Reverse	ATAATTGAGAGTATTTCCATATTAAGCT

**FIGURE 1:**
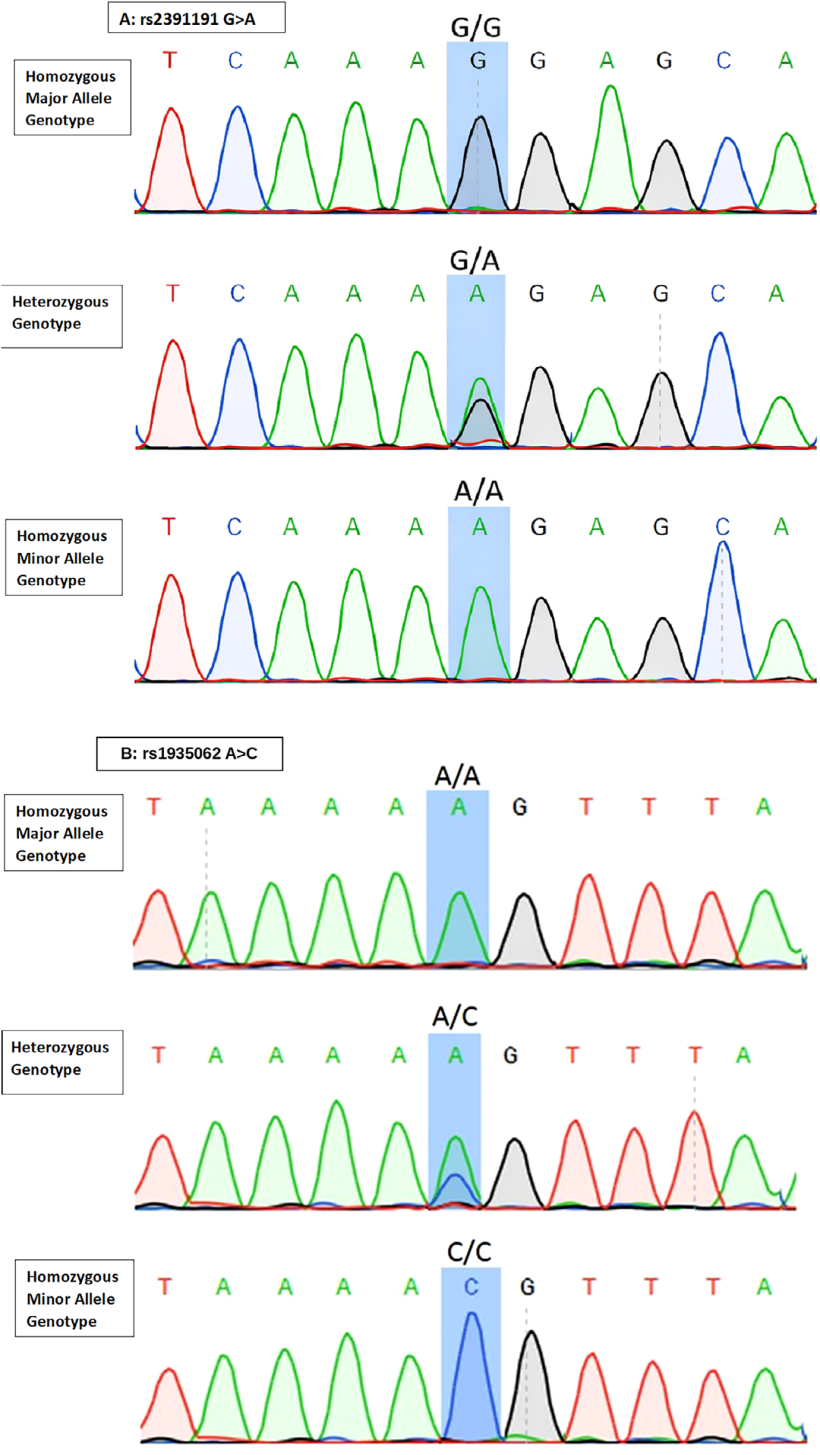
(A) Electropherograms of *DAOA* showing all possible genotypes of rs2391191. (B) Electropherograms of *DAOA* showing all possible genotypes of rs1935062.

In silico PCR and the UCSC genome browser (University of California, Santa Cruz [18]) were used to verify the specificity of primers for the target gene sequences. PCR was performed under standard conditions, followed by gel electrophoresis. Data were collected by visualizing the gel under ultraviolet (UV) light. A subset of randomly selected samples was purified from the gel using QIAGEN MinElute gel extraction kit (Cat. No. 28604). Purified DNA samples were sent to the Keck DNA Sequencing Lab at Yale University, for Sanger sequencing to confirm the specificity of the allele‐specific primers. The electropherograms were visualized using 4Peaks (Figure 1A,B).

### 2.5 Haplotype Analysis

Genotypic data from both SZ and BD patients were analyzed to identify common haplotypes associated with increased risk for these disorders. Haplotype frequency distributions were compared between cases and controls to assess statistically significant associations. Haplotypes for rs2391191 and rs1935062 were inferred from the genotyped data using SNPStats, which applies the expectation‐maximization (EM) algorithm to estimate haplotype frequencies. Pairwise linkage disequilibrium (LD) between the two SNPs was assessed using standard LD metrics D′ and r^2^ to evaluate non‐random allele associations. Haplotypes with a frequency below 1% were excluded from the analysis to maintain robustness. Haplotype‐based associations with SZ and BD were tested using logistic regression models, adjusting for age and sex, with statistical significance defined as *p* < 0.05.

### 2.6 Data Analysis

Differences in age and gender between case and control groups were assessed by applying an independent t‐test and a Chi‐squared test with GraphPad Prism v9. Hardy‐Weinberg equilibrium (HWE) was calculated using the “HardyWeinberg” R package in R v3.3.2. Associations between clinical variables and SNP genotypes were evaluated with Chi‐squared tests in Prism 9.

The relative risk of each SNP for disease association was estimated using an odds ratio (OR) analysis with a 2 × 2 cross‐tabulation and multinomial logistic regression in SAS v9.4 under codominant, dominant, and recessive models. Both unadjusted and adjusted *p*‐values, ORs, and 95% confidence intervals (95% CI) were calculated for each SNP. Adjusted models included age and gender as covariates. A *p*‐value ≤ 0.05 was designated statistically significant.

### 2.7 External Validation: Comparison with GWAS Summary Statistics

To place our findings in the context of large‐scale genetic studies, we compared the allele‐specific associations observed in our cohort with publicly available GWAS summary statistics from the Psychiatric Genomics Consortium (PGC) for SZ and BD [11, 12]. To ensure comparability between datasets, allele orientation was harmonized prior to comparison. In our analyses, the A allele of rs2391191 and the C allele of rs1935062 were defined as the effect alleles. Because GWAS summary statistics may report effect sizes relative to different alleles (commonly denoted as A1), the effect allele reported in each dataset was first identified. When the reported effect allele differed from the allele used in our analysis, the corresponding flipped ORs from the summary statistics were used to evaluate the effect of the same allele analyzed in our cohort. Summary statistics from the latest available PGC SZ and BD GWAS datasets [11, 12] were used for this comparison.

## 3 Results

### 3.1 Patient Characteristics

In the current study, we compared 200 SZ and 120 BD cases with equal numbers of healthy controls from the Pakistani population by genotyping to evaluate the distribution of *DAOA* SNPs and their association with SZ and BD. The average age and gender distribution of patients and controls are presented in Table 2. The SNP IDs and chromosomal location of the *DAOA* gene are presented in Table 3.

**TABLE 2:** Comparison of mean age and gender between healthy controls and patients.

	Controls	SZ	*p*‐value	Controls	BD	*p*‐value
Total Samples	200	200		120	120	
Mean Age ± SD	38.02 ± 17.4	34.28 ± 10.63	0.01^a^	41.6 ± 17.8	32.8 ± 9.7	<0.001^a^
Total Number (Percentage) Males	128 (64%)	130 (65%)	0.83^b^	63 (52.5%)	96 (80.0%)	<0.001^b^
Total Number (Percentage) Females	72 (36%)	70 (35%)		72 (36%)	70 (35%)	

^a^ Calculated using an independent t‐test;

^b^ Calculated using a Chi‐square test.

SZ: schizophrenia

**TABLE 3:** SNP IDs of the *DAOA* gene and their location on chromosomes.

Gene	SNP	Major/Minor allele	Chromosomal location
*DAOA*	rs2391191	G/A	13q33.2
	rs1935062	A/C	

We found a significant difference in age between controls and SZ patients (*p* = 0.01), while no difference was observed for gender (*p* = 0.83); therefore, age was included as a covariate in subsequent statistical analyses for SZ. For BD, a significant difference was found between cases and controls for both age (*p* < 0.001) and gender (*p* < 0.001); therefore, these variables were used as covariates in the BD analysis.

### 3.2 Genotype and Allele Frequency of Studied SNPs

Genotype and allele frequencies of the markers were analyzed across all samples. For *DAOA* SNP rs2391191, the frequency of the homozygous major allele genotype GG was 0.27. Whereas the heterozygous genotype (GA) and homozygous minor allele genotype (AA) showed frequencies of 0.55 and 0.18, respectively. The major allele G had a higher frequency of 0.54, compared to 0.46 for the minor allele A. The global minor allele frequency for A was 0.36.

**FIGURE 2:**
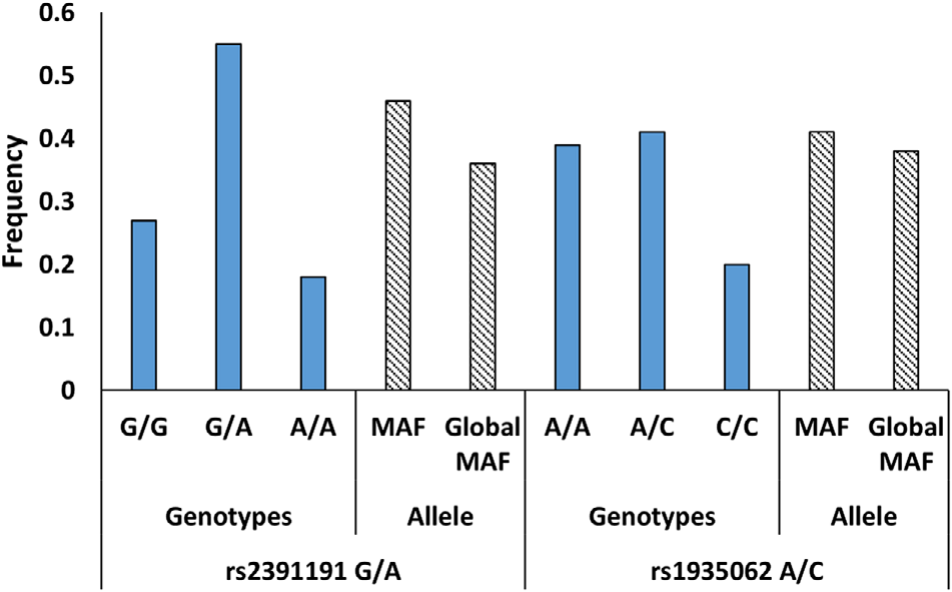
Genotype and allele frequencies of studied SNPs (rs2391191 and rs1935062) in the studied cohort. MAF: Minor allele frequencies.

For *DAOA* SNP rs1935062, genotype frequencies for AA, AC, and CC were 0.39, 0.41, and 0.20, respectively. The major allele A was more frequent (0.59) as compared to minor allele C (0.41) (Figure 2). Global minor allele frequency for C was 0.38. Complete genotype counts for rs2391191 and rs1935062 in BD and SZ cohorts are presented in Table S1, while HWE test results are summarized in Table S2.

### 3.3 Genotypic Association of *DAOA* With Schizophrenia

#### 3.3.1 SNP rs2391191

**FIGURE 3:**
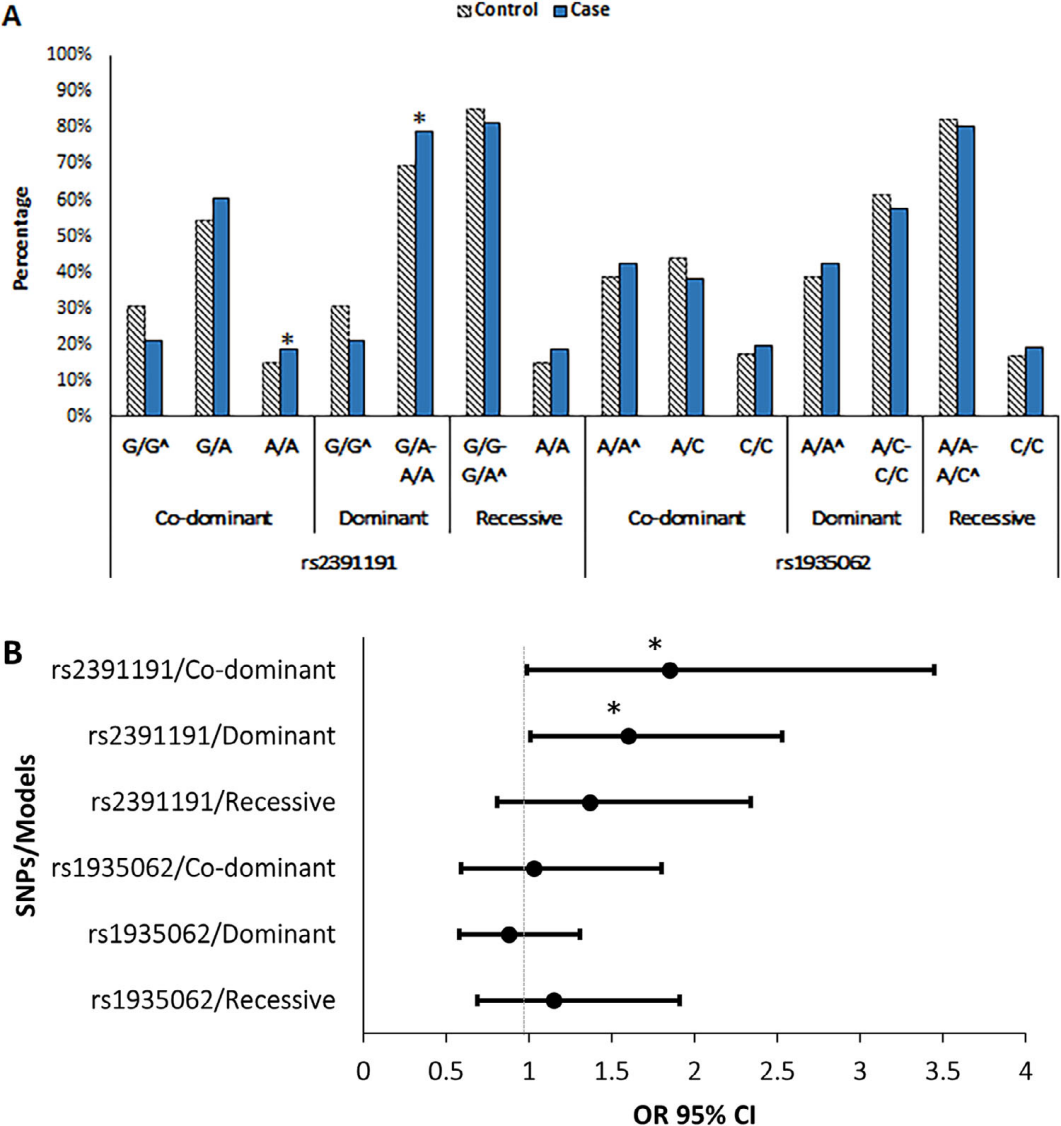
Genotype frequencies of *DAOA* SNPs and associated risk for schizophrenia (SZ) in the studied cohort. (A) Genotype frequencies of *DAOA* variants rs2391191 and rs1935062 in SZ and control groups. Data are presented as percentages. *P*‐values were calculated using multinomial logistic regression. Case group: *n* = 200 individuals with SZ. Control group: *n* = 200 individuals without SZ. ^*^ p ≤ 0.05. ^indicates reference category, ^*^ indicates a significant difference in terms of *p*‐value. For all odds ratios (ORs), the major allele homozygous genotype was used as the reference category. (B) Risk of SZ associated with *DAOA* variants, expressed as odds ratios (ORs) with 95% confidence intervals *(CI)*. The risk estimates were obtained using multinomial logistic regression under codominant, dominant, and recessive models.

Our results showed that for the *DAOA* SNP rs2391191, in the codominant model, 60.5% of cases were heterozygous for the GA genotype, whereas 54.5% of controls showed heterozygosity for the same genotype. The homozygous minor allele genotype (AA) was observed in 18.5% of cases and 15% of controls, in the codominant model (Figure 3A). Under the codominant model, rs2391191 showed a marginal association (OR = 1.87, *p* = 0.05), whereas in the dominant model, rs2391191 was strongly associated with SZ (OR = 1.61, *p* = 0.04) (Figure 3B). However, the recessive model did not show a statistically significant association (OR = 1.39, *p* = 0.22) (Figure 3B). These associations were nominally significant and should be interpreted cautiously in the context of multiple testing. For rs2391191 in the SZ cohort, detailed genotypic association results (adjusted for age and gender) are provided in Table S3.

#### 3.3.2 SNP rs1935062

For the SNP rs1935062, in the codominant model, 38% of cases and 44% of controls were heterozygous for the AC genotype (Figure 3A). The homozygous minor allele genotype CC was observed in 19% of cases and 17.5% of controls. Under the codominant model, rs1935062 did not show an association with SZ (*p* = 0.92 and ORs = 1.03) (Figure 3B). Similarly, no association with disease was observed in the dominant model (p = 0.52, ORs = 0.83), or in the recessive model (*p*‐values = 0.60, ORs = 1.11) (Figure 3B). For rs1935062 in the SZ cohort, the corresponding adjusted genotypic association analyses are also presented in Table S3.

### 3.4 Genotypic Association of DAOA With Bipolar Disorder

#### 3.4.1 SNP rs2391191

**FIGURE 4:**
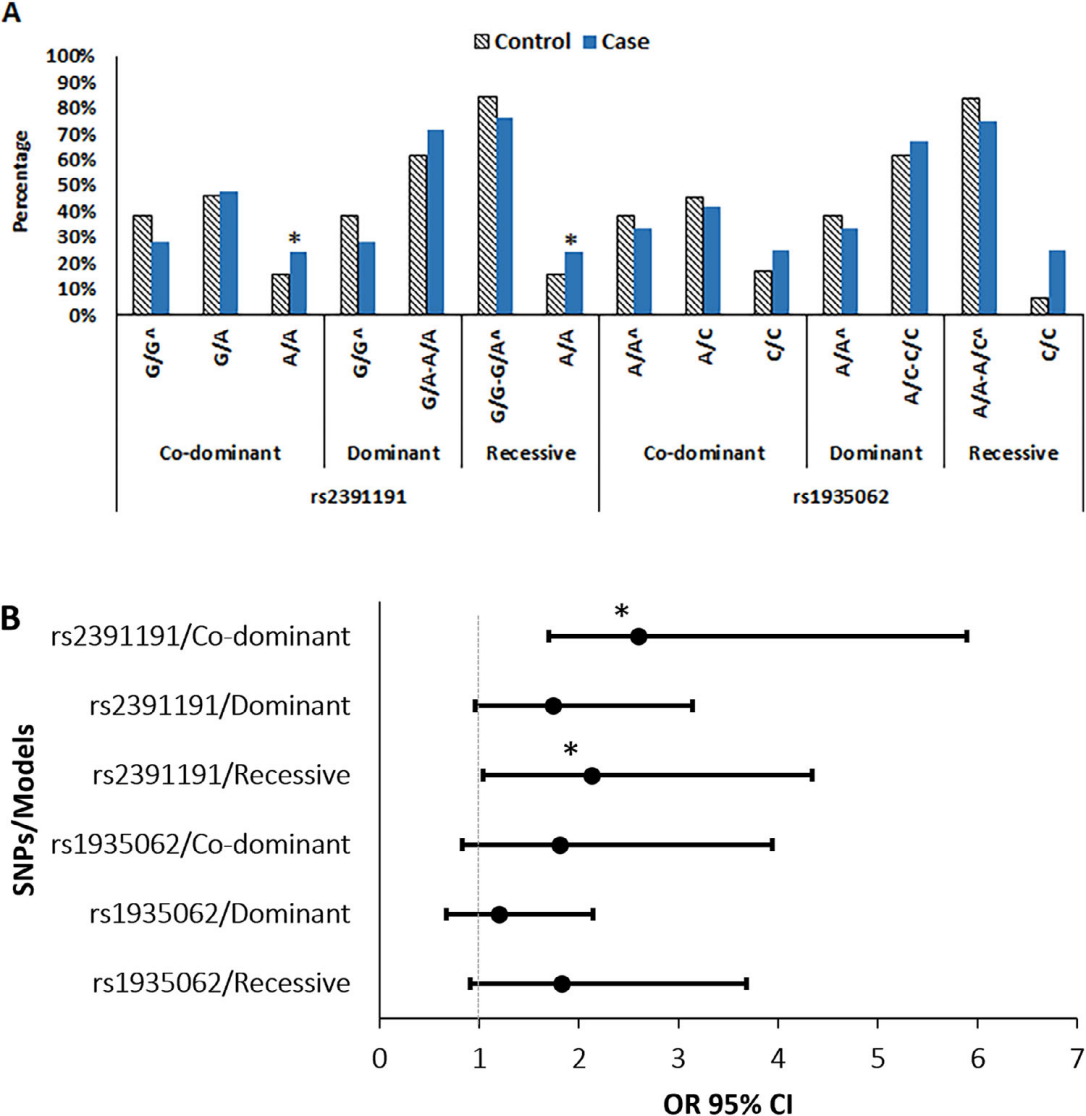
Genotype frequencies of *DAOA* SNPs and corresponding associations for bipolar disorder (BD) in the studied cohort. (A) Genotype frequencies of *DAOA* variants rs2391191 and rs1935062 in BD and control groups. Data are shown as percentages. *p*‐values were calculated using multinomial logistic regression. Case group: *n* = 120 individuals with BD. Control group: *n* = 120 individuals without BD. ^*^ *p* ≤ 0.05. For all odds ratios (ORs), the major allele homozygous genotype was used as the reference category. (B) Risk for BD associated with *DAOA* variants, expressed as odds ratios (ORs) with 95% confidence intervals *(CI)*. Risk estimates were obtained using multinomial logistic regression under codominant, dominant, and recessive genetic models.

The homozygous major allele genotype (GG) of rs2391191 showed frequencies of 28.3% in cases, and 38.3% in controls (Figure 4A). The frequencies observed in the heterozygous genotype (GA) were similar in proportion between cases (47.5%) and controls (45.8%). The minor allele A of homozygous genotype (AA) of rs2391191 was strongly associated with BD in the codominant model (*p* = 0.01, OR = 2.6), as well as in the recessive model (p = 0.03, OR = 2.13) (Figure 4B). However, in the dominant model, the SNP did not show association with BD (*p* = 0.06, OR = 1.74). The observed associations were nominally significant and may be sensitive to multiple testing, warranting cautious interpretation. For rs2391191 in the BD cohort, adjusted genotypic association results are presented in Table S3.

#### 3.4.2 SNP rs1935062

For SNP rs1935062, no significant association was found between genotype frequencies and BD. In the codominant model, the OR for the homozygous minor allele genotype (CC) was 1.81, but the association was not statistically significant (Figure 4B). Similar findings were observed in the dominant and recessive models, where the ORs were greater than 1, i.e., 1.20 and 1.83 respectively, but *p*‐values were non‐significant. For rs1935062 in the BD cohort, the corresponding age‐ and gender‐adjusted genotypic association results are also presented in Table S3.

### 3.5 Identification of Risk and Protective Alleles

**FIGURE 5:**
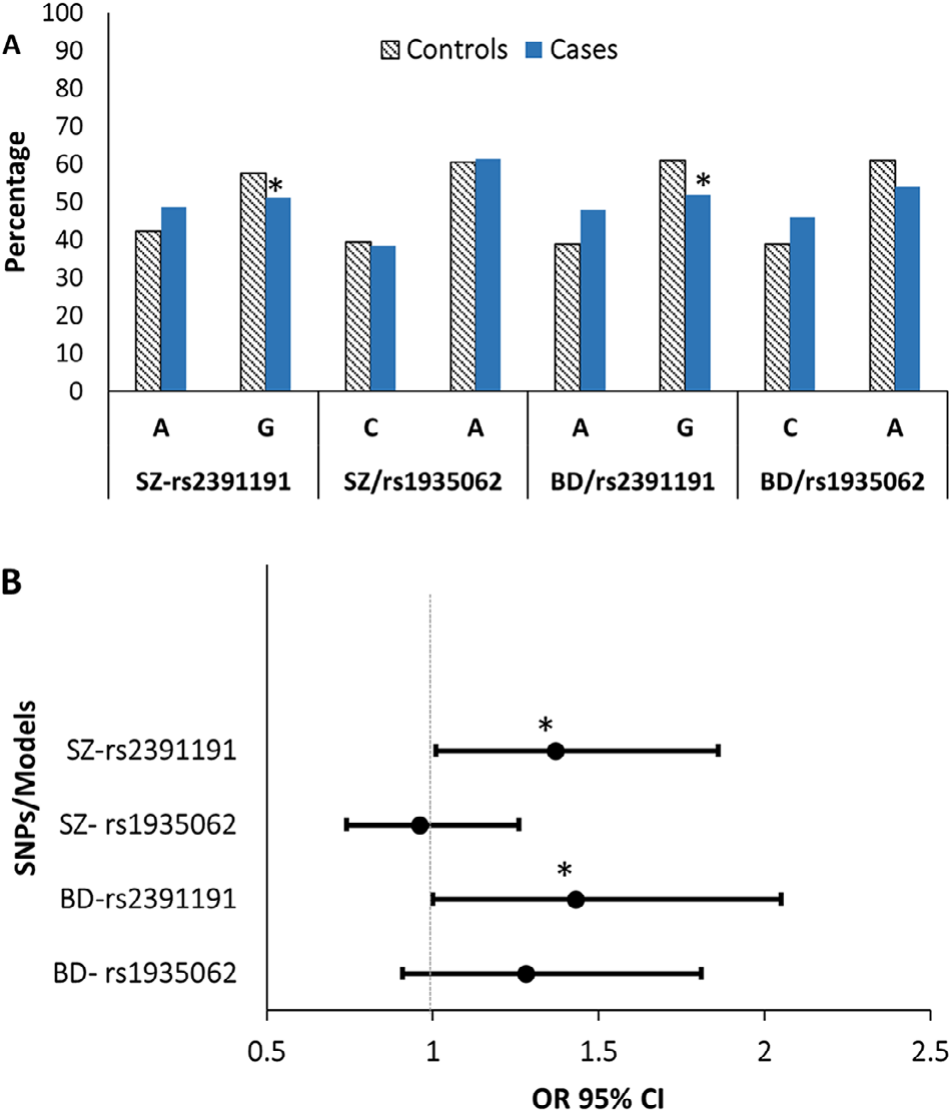
Allele frequencies of *DAOA* SNPs and associated risk for schizophrenia (SZ) and bipolar disorder (BD) in the studied cohort. (A) Allele frequencies of *DAOA* variants rs2391191 and rs1935062 in SZ and BD cases compared with controls. Data are shown as percentages. (B) Risk associated with the *DAOA* alleles, expressed as odds ratio (ORs) with 95% confidence intervals *(CI)*. The risk estimates were obtained using multinomial logistic regression. For allelic odds ratios (ORs), the major allele served as the reference.

*DAOA* SNP rs2391191 of minor allele A was present in 48.7% of cases and 42.3% of controls. We found an OR of 1.38 and a statistically significant *p*‐value of 0.04, suggesting a potential association with SZ in this cohort. For SNP rs1935062, no significant difference was observed in the frequency of the minor allele C between cases (38.5%) and controls (39.5%) (Figure 5A). The observed OR was 0.98 with *p*‐value 0.88, suggesting that the C allele of rs1935062 is not associated with SZ in this cohort (Figure 5B).

A significant allelic difference was observed between BD cases and controls for rs2391191, where the minor allele A was associated with BD (OR = 1.60, *p* = 0.01), suggesting a potential association with BD in the studied Pakistani cohort (Figure 5B). In contrast, no significant difference in allele frequencies was found between BD cases and controls for rs1935062. The minor allele C showed no significant association (OR = 1.29, *p* = 0.18), indicating that it is not associated with BD in this cohort.

### 3.6 Haplotype Analysis

**FIGURE 6:**
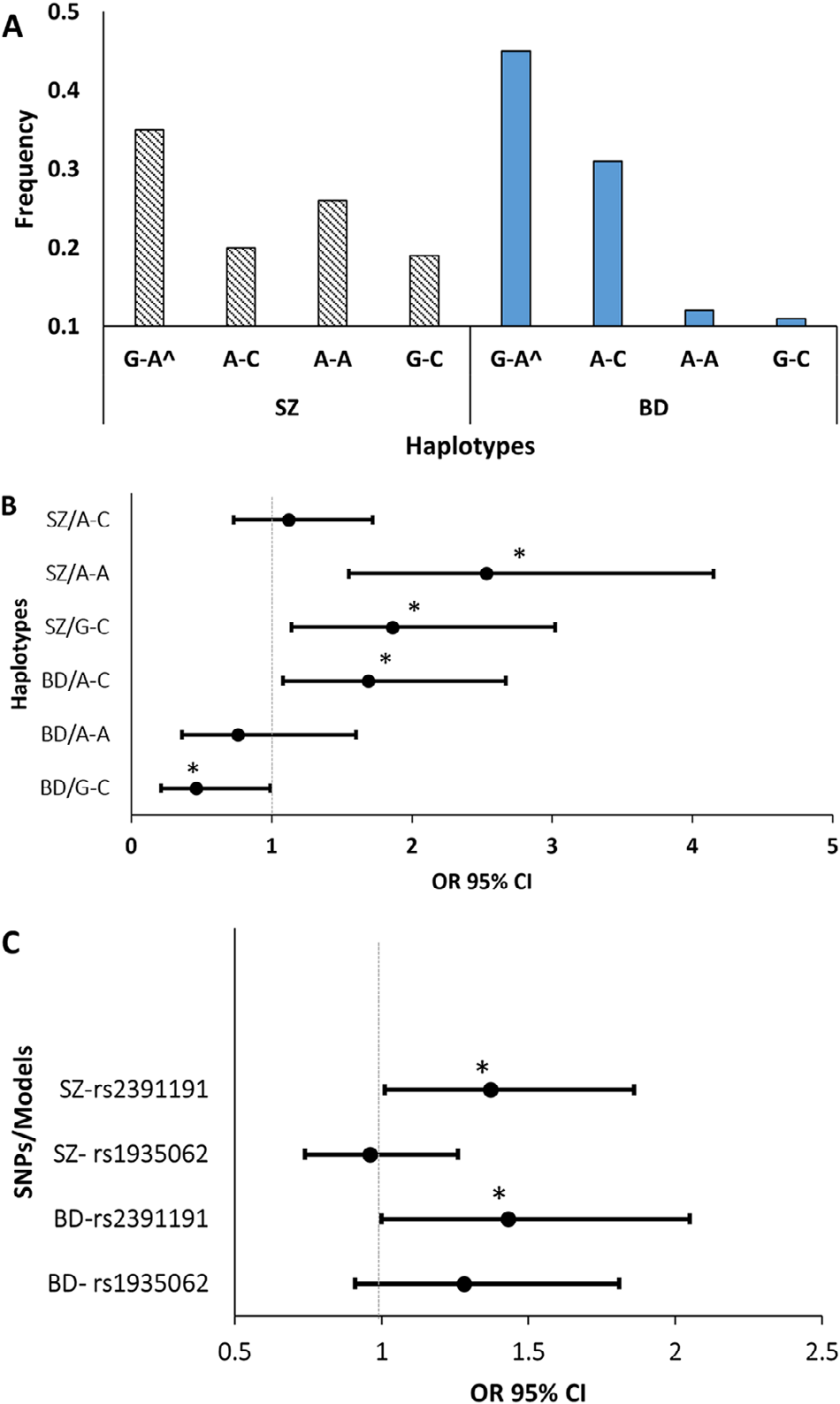
Association of *DAOA* haplotypes with schizophrenia (SZ) and bipolar disorder (BD), and locus‐specific associations between the two disorders. (A) Haplotype frequencies of rs2391191–rs1935062 in SZ and BD. (B) Association of *DAOA* haplotypes with SZ and BD. (C) Locus‐specific associations at the *DAOA* locus between SZ and BD based on allele‐specific associations.

Haplotype analysis of rs2391191–rs1935062 indicated that the A‐A haplotype showed an association with SZ (OR = 2.53, p = 0.0003). However, this finding should be interpreted cautiously in the context of multiple testing, given that formal correction for multiple comparisons was not applied. The G‐C haplotype was associated with SZ, with an OR of 1.86 and a *p*‐value of 0.01. In the case of BD, the A‐C haplotype was significantly associated with BD, with an OR of 1.69 and a *p*‐value of 0.02 (Figure 6A,B). Although statistically significant at the nominal level, these associations may not remain significant after correction for multiple comparisons. Pairwise LD analysis between rs2391191 and rs1935062 is presented in Table S4. Haplotype‐based association analyses with disease status, adjusted for age and gender, are presented in Table S5.

### 3.7 Locus‐Specific Associations at the DAOA in Schizophrenia and Bipolar Disorder

The estimation of risk and protective alleles for rs2391191 and rs1935062 at the *DAOA* locus in both SZ and BD indicated locus‐specific associations. The A allele of rs2391191 showed nominal associations with both disorders (OR = 1.38, *p* = 0.04 for SZ; OR = 1.60, *p* = 0.01 for BD).

In contrast, for rs1935062, the observed ORs were 0.98 for SZ and 1.29 for BD, with *p*‐values 0.88 and 0.18, respectively, indicating no significant association with either disorder (Figure 6C).

### 3.8 Comparison of Our Findings With Psychiatric Genomics Consortium (PGC) Genome‑Wide Association Study (GWAS) Data

For rs1935062, the C allele showed no significant association with either SZ or BD in our cohort. Similarly, PGC GWAS datasets did not report significant associations for this variant across multiple ancestral populations. In contrast, rs2391191 showed significant association with both BD and SZ in our cohort; however, PGC GWAS datasets indicate that the association with BD is primarily driven by the alternate G allele, which shows significant effects in several bipolar datasets. For SZ, GWAS datasets do not report a significant association for this variant; however, when interpreted relative to the A allele, a weak trend toward increased risk that does not reach statistical significance was observed. Comparative analysis of allele‐specific effects between our cohort and PGC GWAS datasets, with effect alleles aligned across studies, is presented in Table S6.

### 3.9 Association of Genotypes With Clinical Variables of Schizophrenia and Bipolar Disorder

Eight clinical variables for SZ and seventeen for BD were documented for each patient to further examine the association of selected candidate SNPs with the behavioural features of SZ and BD, respectively.

For SZ, the clinical features “feelings of threat from others” (*p* = 0.001), “restricted mood” (*p* = 0.03), and “lack of self‐care skills” (*p* = 0.03) showed nominal associations with rs1935062. No clinical variables in SZ were found to be associated with rs2391191 (Table 4). For BD, the clinical phenotype of insomnia and disorganized thoughts showed nominal association with rs1935062 of *DAOA* (*p* = 0.010, and *p* = 0.03, respectively) (Table 5). These findings should be interpreted cautiously, as they were identified in the absence of an overall disease association and may reflect chance findings arising from multiple comparisons.

**TABLE 4:** Association of genotypes with clinical variables of schizophrenia.

	*DAOA rs1935062*		*DAOA/rs2391191*	
Clinical variables	Chi‐Sq	*p*‐value	Chi‐Sq	*p*‐value
Gender	5.50	0.064	0.13	0.934
Auditory hallucination	4.20	0.122	0.78	0.675
Unusual experience	3.47	0.176	0.97	0.615
Feelings of threat from others	13.26	**0.001**	0.67	0.714
Restricted mood	6.57	**0.037**	1.48	0.476
Inability to self‐care	7.04	**0.030**	2.54	0.280
Aggressive behavior	2.25	0.323	3.47	0.176
Suspicious behavior	1.75	0.417	0.86	0.649
Poor social functioning	4.29	0.117	2.37	0.306

**TABLE 5:** Association of genotypes with clinical variables of bipolar disorder.

	*DAOArs1935062*		*DAOArs2391191*	
Clinical variables	Chi‐Sq	*p*‐value	Chi‐Sq	*p*‐value
Inflated self‐esteem	5.53	0.063	4.80	0.091
Decreased need for sleep	1.94	0.379	0.11	0.942
Increased talkativeness	0.14	0.931	0.67	0.716
Disorganized thoughts	6.82	**0.033**	1.34	0.513
Distractibility	0.63	0.727	1.18	0.555
Increased involvement in goal‐directed behaviour	0.36	0.835	2.27	0.321
Increase in risky behaviour	1.38	0.501	1.67	0.433
Fatigue	0.42	0.809	1.93	0.380
Feelings of worthlessness or guilt	2.81	0.245	1.35	0.508
Difficulty in thinking, concentrating, making decisions	2.17	0.337	0.68	0.711
Recurrent thoughts of death or suicide, plans, or attempts	0.42	0.810	2.30	0.316
Depressed mood all day	1.29	0.525	2.78	0.249
Diminished interest in daily activities	0.63	0.730	4.62	0.099
Change in weight or appetite	1.80	0.406	0.63	0.728
Insomnia	9.29	**0.010**	3.26	0.195
Hypersomnia	2.35	0.308	2.69	0.260
Psychomotor retardation or agitation	0.75	0.687	3.46	0.177

## 4 Discussion

The present study explored the association of *DAOA* SNPs rs2391191 and rs1935062 with schizophrenia (SZ) and bipolar disorder (BD) in a Pakistani cohort. The results of genotypic association analyses of *DAOA* variants suggested a nominal association of rs2391191 with both SZ and BD, whereas the C allele of rs1935062 did not show a consistent disease association. Specifically, rs2391191 exhibited significant genotype associations under codominant and dominant models in SZ and under codominant and recessive models in BD, while rs1935062 lacked significant genotype associations in either disorder.

Allelic analyses reinforced the genotype findings: the A allele of rs2391191 was significantly more frequent in both SZ and BD cases compared to controls, suggesting a possible role in SZ and BD within the studied cohort. This is consistent with meta‐analytic evidence implicating rs2391191 in SZ susceptibility [7]. In addition, rs2391191 AA homozygotes have been shown to exhibit reduced white matter integrity in first‐episode SZ patients [19], and altered prefrontal cortical function [20], further supporting its neurobiological relevance. However, associations with BD have been inconsistent, with some studies reporting no significant relationship [21], suggesting potential population‐specific or phenotype‐specific effects. The rs2391191 variant was also reported to be associated with SZ by Chen and colleagues [22].

Conversely, our findings differ from previous studies such as Chiesa et al. (2011), where no association was found between rs2391191 in *DAOA* and SZ in European and Asian populations [21, 23]. Furthermore, several other studies conducted in European populations also failed to find any evidence supporting an association between *DAOA* and SZ [24, 25, 26]. The present study found no significant differences in genotypic or allelic frequencies of rs1935062 in either SZ or BD.

These inconsistencies in findings across different populations may be explained by allelic heterogeneity, which is also evident from our results showing differences in globally reported major and minor allele frequencies (MAF) compared to the studied Pakistani population. The absence of significant allelic association for rs1935062 in our cohort emphasizes the complex population‐specific nature of *DAOA* allelic effects. Such allelic heterogeneity may arise from differences in local LD patterns or interaction with other genetic and environmental factors that vary by ancestry.

Haplotype analyses provided additional insight into multi‐locus effects. In SZ, the A‐A and G‐C haplotypes spanning rs2391191–rs1935062 were significantly associated with disease, while in bipolar disorder, the A‐C haplotype was significantly associated. This suggests that specific allele combinations may exert additive or interactive effects beyond single marker associations. Previous *DAOA* haplotype studies have yielded mixed results. Some studies demonstrated significant haplotype associations across the *DAOA* locus in SZ [27], with subsequent studies identifying haplotype effects in both SZ and BD [28]. However, larger replication studies in European cohorts did not detect consistent haplotype associations, and additional analyses highlighted ethnic variability in haplotype structure and disease correlation [29, 30]. These discrepancies highlighted the importance of considering haplotype context and ethnic diversity when interpreting *DAOA* locus effects. Given the number of comparisons performed across SNP‐level, haplotype, and genotype‐phenotype analyses, these findings should be regarded as exploratory and interpreted with appropriate caution and require replication in independent cohorts.

In terms of genotype‐phenotype associations, rs1935062 was nominally associated with insomnia and disorganized thoughts in BD patients, as well as with restricted mood, feelings of threat from others, and reduced self‐care skills in SZ patients. While rs1935062 did not show any association with overall disease susceptibility in our cohort, these findings should be interpreted with considerable caution. In the absence of a primary disease association and given the number of phenotype comparisons performed, these associations may reflect chance findings rather than true biological effects. At most, they may tentatively suggest a possible phenotype‐modifying role; however, this remains unconfirmed and requires replication in independent cohorts. Therefore, rs1935062 should not be considered a susceptibility variant based on the current data. While symptom domains such as insomnia and disorganized thinking are well documented in psychiatric disorders [31, 32, 33], the present findings do not provide sufficient evidence to support a specific mechanistic link. These features are also consistent with prior literature linking threat perception and negative emotional states to cognitive dysfunction in psychiatric disorders [34]; however, the present findings do not establish a direct relationship between rs1935062 and these processes.

Studies have also shown association of rs2391191 with impaired cognitive performance, a mixed phenotype of psychosis and mania, and deficits in neuropsychological traits [35]. Additionally, rs2391191 has been reported to be linked with reduced fractional anisotropy, indicating impaired white matter integrity in the corpus callosum of SZ patients [19]. Moreover, overexpression of *DAOA* in SZ patients has been observed in the dorsolateral prefrontal cortex compared to healthy controls [36]. The rs2391191 polymorphism has also been associated with cortical thinning in risk allele carriers. However, in our study, no significant genotype‐phenotype associations were observed for rs2391191 in either SZ or BD patients.

While rs1935062 did not show a significant association with overall disease susceptibility in our cohort, its exploratory associations with specific clinical phenotypes should be interpreted cautiously and remain unconfirmed. In complex psychiatric disorders, genetic heterogeneity is well recognized, and susceptibility loci may differ from variants that influence symptom dimensions, severity, or clinical subtypes. As argued by Fanous and Kendler, modifier genes can shape quantitative traits and clinical manifestations independently of core disease liability, contributing to phenotypic variability within diagnostic categories [37]. Within this framework, rs1935062 is unlikely to act as a major susceptibility locus and, if anything, could at most tentatively modulate specific psychiatric features once disease is present, though this possibility remains unconfirmed. This interpretation aligns with models of complex traits suggesting that genetic modifiers may play a significant role in psychiatric disorders, and importantly, phenotypic expression can be influenced by variants outside of the primary disease‐causing gene [38, 39]. These observations, however, remain hypothesis‐generating and require replication in independent cohorts before any firm conclusions can be drawn.

The differences observed across populations may be explained by the fact that these disorders exhibit a heterogeneous pattern of expression, with different loci influencing disease liability in different populations. Furthermore, individual SNPs may carry varying levels of informational content, and it is expected that they exhibit unstable frequencies across populations, particularly in association studies of complex disorders. Overall, risk haplotypes within *DAOA* have previously been found to correlate significantly with negative symptoms, depressive features, and cognitive impairment in SZ, supporting the hypothesis that genetic variation in *DAOA* may contribute to specific clinical characteristics of SZ [40].

To further contextualize the associations observed at the *DAOA* locus, we compared our findings with results from large‐scale genome‐wide association studies conducted by the PGC for SZ and BD [11, 12]. The comparison indicated that rs1935062 did not show consistent association with either SZ or BD across large international GWAS datasets, which is consistent with the absence of association observed in our cohort (SZ: OR = 0.98, *p* = 0.88; BD: OR = 1.29, *p* = 0.18). This concordance further reinforces that rs1935062 is unlikely to represent a primary susceptibility variant for either disorder; any residual interest in this variant should therefore be confined strictly to its potential exploratory relevance to specific symptom dimensions, pending replication.

In contrast, rs2391191 showed a nominal association with both SZ and BD in the Pakistani cohort (SZ: OR = 1.38, *p* = 0.04; BD: OR = 1.60, *p* = 0.01), whereas large GWAS datasets generally reported only very small effect sizes for this variant. These findings suggest that the contribution of rs2391191 to disease susceptibility may vary across populations. Differences in allele frequencies, local LD structure, or broader genetic background may influence how variants within the *DAOA* locus contribute to disease risk in different ancestral groups. Such population‐dependent effects are well recognized in psychiatric genetics, where variants with modest effects in large multi‐ancestry studies may appear stronger in specific populations due to differences in genetic architecture or local haplotype structure. The stronger effect observed in the present study may therefore reflect population‐specific genetic influences at the *DAOA* locus or stochastic variation related to the moderate sample size.

**FIGURE 7:**
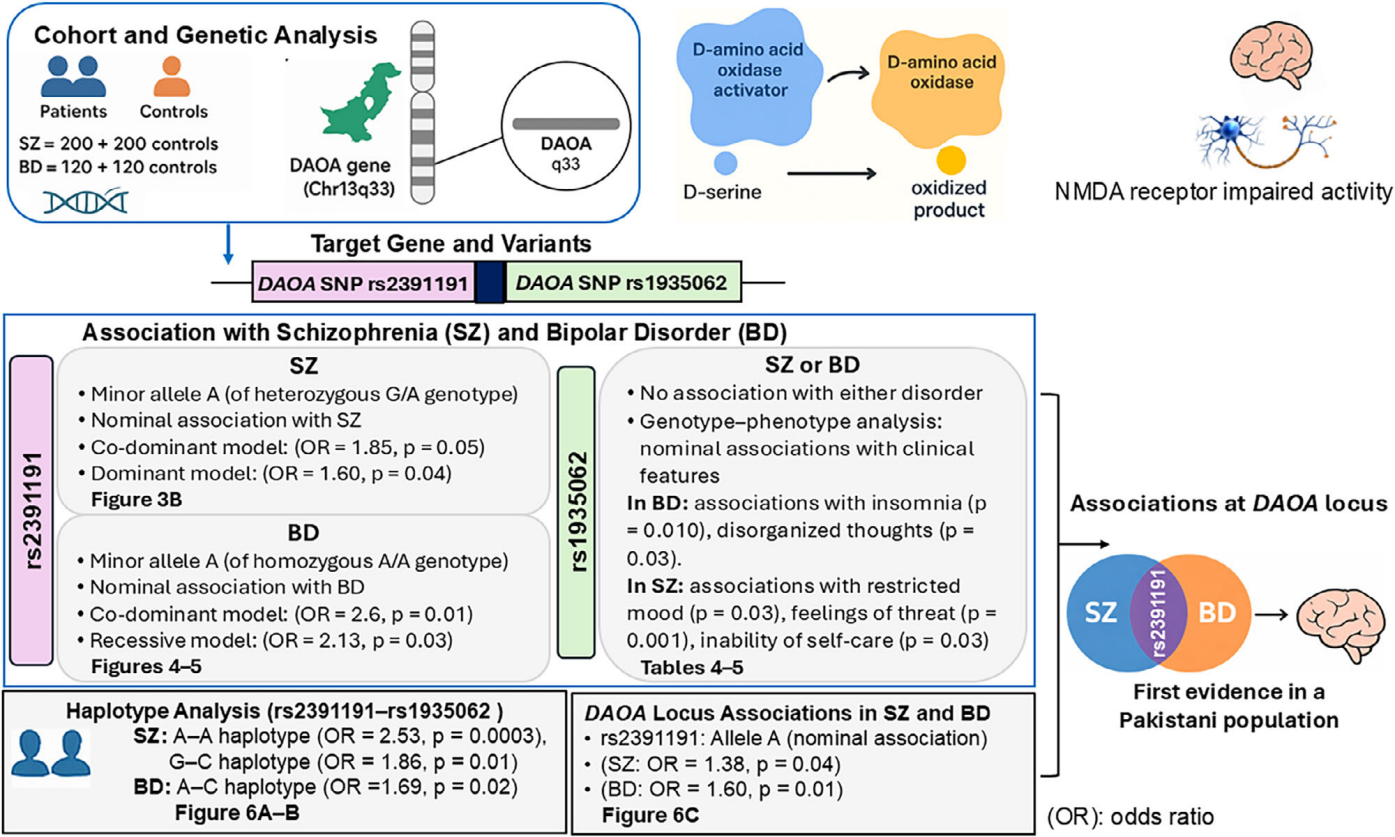
Schematic overview of locus‐specific genetic associations at the *DAOA* gene in schizophrenia (SZ) and bipolar disorder (BD). Shown are the study design, including cohort characteristics, selection of *DAOA* SNPs (rs2391191 and rs1935062), and downstream analyses comprising genotype, allele, haplotype, and genotype‐phenotype associations. The variant rs2391191 showed a nominal association with both SZ and BD, whereas rs1935062 showed no association with overall disease risk but demonstrated exploratory associations with selected clinical features. These findings are exploratory and require replication in independent cohorts.

A key strength of the present study is the evaluation of locus‐specific genetic variation within the *DAOA* gene in both SZ and BD within the same population, allowing direct assessment of locus‐specific associations at the *DAOA* locus (Figure 7). In addition, the integration of genotype, haplotype, and clinical phenotype analyses provides a parallel evaluation of the potential role of *DAOA* variants in these disorders. Furthermore, comparison of our findings with publicly available GWAS datasets from the PGC [11, 12] allowed us to contextualize the observed associations within the framework of large‐scale international genetic studies.

However, the present study has some limitations. First, risk association was assessed using only two SNPs within a single gene locus (*DAOA*), which restricts the scope of inference. In addition, given the number of comparisons performed across SNP‐level, haplotype, and genotype‐phenotype analyses, the possibility of type I error cannot be excluded. Importantly, formal correction for multiple testing was not applied, and therefore some of the nominally significant associations, particularly those with modest effect sizes, may not remain significant after such correction. Accordingly, all findings, including those observed for rs2391191, should be interpreted as exploratory and hypothesis‐generating rather than definitive evidence of association. Furthermore, the moderate sample size limits statistical power and may contribute to variability in effect estimates. Future studies incorporating larger cohorts, additional polymorphisms across the *DAOA* locus, and genome‐wide approaches, together with appropriate correction for multiple comparisons, will be necessary to determine the robustness and generalizability of these findings.

## 5 Conclusion and Outlook

The current study provides preliminary, locus‐specific observations suggesting that variation at the *DAOA* gene may be associated with SZ and BD in a Pakistani cohort. The observed association of rs2391191 with both disorders should be interpreted cautiously, as the analysis was limited to two variants and did not include correction for multiple testing. In contrast, rs1935062 showed no association with overall disease risk, and its nominal associations with clinical features should be regarded as exploratory and potentially attributable to chance.

To the best of our knowledge, this study represents the first report demonstrating exploratory locus‐specific associations of *DAOA* variants with both SZ and BD in a Pakistani cohort, providing a foundation for future genetic investigations in this population. However, given the candidate‐gene design, modest sample size, and limited genomic scope, the present study does not support inference regarding broader shared genetic architecture or underlying biological pathways between SZ and BD. Rather, the results are restricted to locus‐specific observations within *DAOA* and should be considered hypothesis‐generating. Further studies in larger, independent cohorts, incorporating genome‐wide approaches and appropriate correction for multiple comparisons, will be required to determine the robustness and generalizability of these observations.

## Author Contributions

Conceptualization, G.K.R. J.L. and M.K.; methodology, M.K., G.K.R. M.N. and J.L.; software, M.K., A.S., M.F.A., G.K.R.; validation, M.K., G.K.R., T.F., and M.N.; formal analysis, M.K., M.S., G.K.R., and M.N.; investigation, M.K., G.K.R., and M.N.; resources, J.L., G.K.R. and M.N.; data curation, M.K, A.N, and G.K.R.; writing – original draft preparation, M.K., A.S., G.K.R.; writing – review and editing, M.K., M.M., M.S., A.S., S.M.B., A.N., S.M., M.F.A., T.F., F.F., J.L., M.N. and G.K.R.; visualization, M.K., G.K.R. and M.N.; supervision, J.L., G.K.R. and M.N.; project administration, J.L., G.K.R.; funding acquisition, J.L., M.K., G.K.R. All authors have read and agreed to the final version of the manuscript.

## Funding

Higher Education Commission of Pakistan under the “International Research Support Initiative Program” PIN IRSIP 33 BMS 07

## Conflicts of Interest

The authors declare no conflicts of interest.

## Supporting information




**Supporting File**: ggn270038‐sup‐0001‐TableS1‐S6.xlsx.

## Data Availability

The data that support the findings of this study are available from the corresponding author upon reasonable request. A supplementary data file containing the complete genotype counts, HWE test results, LD metrics (D′ and r^2^), full haplotype association statistics, and the PGC GWAS comparison values are available at https://doi.org/10.5281/zenodo.20055652.
